# Monitoring of circulating tumor DNA and its aberrant methylation in the surveillance of surgical lung Cancer patients: protocol for a prospective observational study

**DOI:** 10.1186/s12885-019-5751-9

**Published:** 2019-06-13

**Authors:** Guannan Kang, Kezhong Chen, Fan Yang, Shannon Chuai, Heng Zhao, Kai Zhang, Bingsi Li, Zhihong Zhang, Jun Wang

**Affiliations:** 1 Peking University People’s Hospital, Beijing, People’s Republic of China; 2 Burning Rock Biotech, Guangzhou, People’s Republic of China

**Keywords:** Lung cancer, Circulating tumor DNA, DNA methylation

## Abstract

**Background:**

Detection of circulating tumor DNA (ctDNA) is a promising method for postoperative surveillance of lung cancer. However, relatively low positive rate in early stage patients restricts its application. Aberrant methylation of ctDNA can be detected in blood samples, and may provide a more sensitive method. This study is designed to systematically evaluate and compare the detection of aberrant methylation and mutations in ctDNA among surgical non-small cell lung cancer (NSCLC) patients, aiming to investigate the feasibility of ctDNA detection as a means of lung cancer surveillance.

**Methods:**

This is a prospective observational study. Consecutive surgical NSCLC patients will be recruited. Blood samples will be collected both before and after surgery (during the follow-up period), while matching tumor tissues and tumor-adjacent normal tissues will be collected during surgery. Quantitative analysis of aberrant methylation and mutations of ctDNA will be conducted in combination with a three-year follow-up data.

**Discussion:**

This is the first registered prospective study designed to investigate the feasibility of ctDNA methylation detection as a means of postoperative lung cancer surveillance. We will systematically evaluate and compare the quantitative detection of ctDNA mutations and ctDNA methylation in surgical NSCLC patients, combining with the follow-up information. By integrating genetic and epigenetic information of ctDNA, more effective strategies for postoperative surveillance may be defined.

**Trial registration:**

This study (MEDAL, MEthylation based Dynamic Analysis for Lung cancer) was registered on ClinicalTrials.gov on 08/05/2018 (NCT03634826; Pre-results).

## Background

Lung cancer is the leading cause of cancer related death worldwide [1]. Although some early stage patients can get long-term disease free survival after curative surgery, there are still 20–40% stage I/II patients who suffer local recurrence or distant metastasis [2]. Patients with the same TNM stage may achieve greatly differential outcomes, indicating there are heterogeneities and traditional staging system might not be adequate. For postoperative patients, clinical visits and CT (Computed Tomography) are recommended as a principal means of tumor supervision [3]. However, although CT scans could detect disease progression earlier than X-ray for postoperative patients, overall survival of the two groups showed no statistical significance, indicating that traditional radiological methods may have already reached the limit and this probably due to the delay of early diagnosis and treatment. How to identify minimal residual disease (MRD) after curative surgery, as well as predict and detect recurrence without delay, still remains elusive.

Circulating tumor DNA (ctDNA) is a kind of tumor-specific DNA derived from tumor cells, which in principle contains the same genetic information of tumor tissue [4]. Detection of ctDNA with peripheral blood samples has been reported in different tumor types, especially in patients with advanced stage [5–7]. The eliminating rate of ctDNA is relatively high, making it an ideal biomarker to reflect tumor burden and providing a potential method for tumor management [4, 8–10]. In the field of non-small cell lung cancer (NSCLC), ctDNA has already shown its significance and gradually been recognized as a way to guide clinical practice [11–13]. However, previous researches focused mainly on patients of advanced stage (stage IIIB to IV). Studies of surgical patients (stage IA to III) were relatively rare, and data from early stage patients (stage IA to IIB) was especially scarce [14–16]. In addition, the amount of ctDNA is extremely low in early stage patients, increasing the difficulty of detection by sequencing, especially when there is a large amount of background cell free DNA (cfDNA) [4]. Although some institutions have explored the possibility of MRD detection after surgery, the sensitivity of ctDNA in early stage patients is not satisfying [17, 18]. In the recently published TRACERx study, the positive rate of ctDNA detected in stage I lung adenocarcinoma was only 19% [17]. To overcome these limitations, exploring multi-omics data may provide a promising strategy, such as integrating genetic and epigenetic information of ctDNA.

Methylation is one of the most common epigenetic alterations [19]. Methylation of CpG dinucleotide-rich clusters of gene promoter regions can cause gene silencing, which plays a significant role in the initiation and progression of malignant tumors.^6 19^ Previous studies indicated that aberrant methylation of DNA can be a promising biomarker. Brock et al. [20] illustrated a panel of 4 aberrant methylated genes, which were related to the recurrence of postoperative lung cancer patients. Recently, Akira et al. [21] demonstrated a different gene panel of methylation which could predict prognosis of NSCLC. In addition, aberrant methylation is often an early event in carcinogenesis and is detectable in blood samples [4]. With limited published researches, it seems that detection of methylated ctDNA can be a more sensitive method in early stage NSCLC patients [21]. However, most previous studies focused on the methylation of tumor tissues, while methylation of ctDNA in blood samples hasn’t been well illustrated [19, 20]. In the field of lung cancer, researchers explored different candidate genes or gene combinations, while an optimal panel of methylation still remains to be discovered [20, 21]. Besides, although ctDNA methylation has shown its potential of diagnosis and prognostic prediction value in several studies, some basic characteristics of ctDNA methylation still remains to be confirmed by prospective studies.

Conclusively, detection of ctDNA mutations as well as aberrant ctDNA methylation can be a promising way for the surveillance of NSCLC, and calls for further studies.

### Previous work

The detection of ctDNA in late stage lung cancer has been well illustrated by previous studies. To evaluate the feasibility of ctDNA detection in surgical NSCLC patients, we enrolled 76 NSCLC patients who underwent curative-intent lung resection and analyzed their blood samples as well as tumor tissues using a 50 cancer related mutation panel. The overall concordance rate between plasma samples and tissue samples was 68.4%, and would increase according to TNM stage (stage I, 57.9%; stage II, 66.7%; stage IIIA, 90%, *P* = .043). Additionally, ctDNA was more sensitive compared with traditional tumor markers, and has higher predictive value than classic prediction models.^11 12^

Furthermore, we explored the stability and dynamic changes of ctDNA in surgical lung cancer patients. Plasma samples were obtained before (1 to 3 days) and during surgery for 20 patients. All ctDNA samples obtained before and during surgery had consistent mutations, and the mutation frequency varied at a very low level, indicating that plasma ctDNA offers reliable and consistent information. As dynamic changes of ctDNA in lung cancer patients remained to be clarified, we explored its half-life period and confirmed a rapid clearance after surgery. Three days after surgery was considered as an optimal start time for recurrence monitoring, as ctDNA concentration would reduce to zero theoretically. Interestingly, during the follow-up of postoperative patients, we found that ctDNA detection was in conformity with patients’ outcome [11, 12, 22].

To sum up, we have completed a series of studies in the field of liquid biopsy, demonstrated the feasibility of ctDNA detection in surgical NSCLC patients, and shown its potential value for postoperative surveillance.

### Hypotheses

1. For stage IA to III surgical NSCLC patients, blood samples and tumor tissues have high concordance for both DNA mutation and aberrant DNA methylation.
2. Detection of ctDNA methylation has comparable, if not greater, sensitivity than that of ctDNA mutation in early stage patients.
3. Quantitative changes of ctDNA mutation and its aberrant methylation can be used to predict patients’ prognosis, and can reflect tumor progression earlier than radiological examinations.

### Primary aims

1. To investigate the relationship between surgical lung cancer patients’ prognosis and quantitative changes in ctDNA mutations and aberrant methylation.
2. To investigate the variation of aberrant methylated ctDNA concentration before surgery, 3 days after surgery and 1 month after surgery.

### Secondary aims

1. To analyze the conformity of DNA mutation and aberrant DNA methylation between blood samples and tumor tissues of stage IA to III surgical patients.
2. To illuminate the characteristics and relationship of tumor DNA methylation, ctDNA methylation, and tumor-adjacent normal tissues methylation.
3. To classify stage IA to III surgical patients by genetic changes and signaling pathway changes, and to explore the relationship between molecular characteristics and postoperative DFS.
4. For patients who receives adjuvant therapy, to investigate the consistency of postoperative DFS and the variation in abundance of ctDNA mutations or aberrant methylation.
5. To evaluate whether detection of ctDNA methylation and mutations can predict tumor progression earlier than radiological examination in some patients, and illustrate its leading time.

### Methods/design

**Fig. 1:**
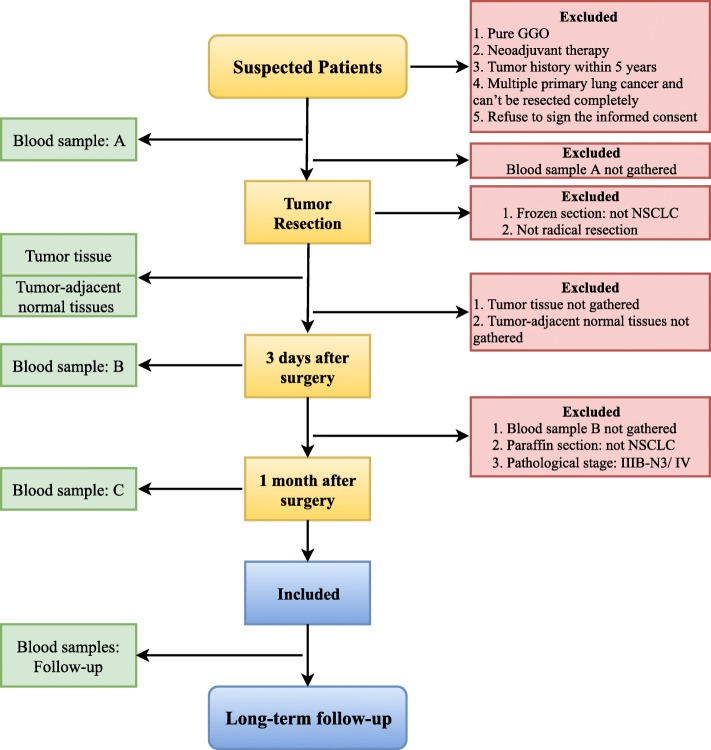
Flow chart of patient recruitment, sample collection and follow-up

This protocol outlines a prospective observational study (MEDAL, MEthylation based Dynamic Analysis for Lung cancer) in which surgical NSCLC patients will be recruited. Blood samples will be taken before and after surgery, while tumor tissue samples and tumor-adjacent normal tissues will be collected during surgery. Patients recruited will be followed for at least three years after surgery (Fig. 1).

### Study population

The population will be recruited from Peking University People’s Hospital Thoracic Surgery Department. All of the patients will receive thoracic CT scans (with or without contrast), abdominal and adrenal gland ultrasonography or CT, brain MRI (Magnetic Resonance Imaging) or CT and radionuclide bone scan before surgery. Positron Emission Tomography (PET/CT) will not be mandatory. Traditional tumor markers will be examined, including carcinoembryonic antigen (CEA), carbohydrate antigen 125 (CA-125), carbohydrate antigen 199 (CA-199), cytokeratin 19 fragment (CYFRA21-1), neuron-specific enolase (NSE), and interleukin 6 (IL-6). Consecutive patients who are suspected stage IA- III NSCLC before surgery will be eligible and included in this study. Patients who will be enrolled in this study should meet the following conditions: (1) Radiology examinations suspects lung cancer, and patients who will be treated by curative surgery. (2) Suspected stage between IA to III. (3) Understand and agree with the informed consent. The exclusion criteria are as followed: (1) Patients with pure ground glass opacity (pGGO). (2) Have received chemotherapy, radiotherapy, targeted therapy, or immunotherapy before surgery. (3) Malignant tumor history within the past 5 years. (4) Multiple primary lung cancer and can’t be resected completely. (5) Pathology of frozen sections or paraffin sections proves the tumor is not NSCLC. (6) Pathological stage proves to be IIIB-N3 or IV. (7) Samples of blood or tissues are unqualified or cannot be obtained. (8) Refuse to sign the informed consent.

Follow-up information will be obtained 1 month after surgery and every 3–6 months thereafter by an experienced thoracic surgeon. The follow-up protocol consists of chest CT scan and abdominal ultrasound performed every 6 months for the first 2 years and yearly thereafter. Traditional tumor markers will be examined every 6 months. Brain MRI or CT and radionuclide bone scan will be performed when patients have symptoms.

Clinical and demographic data will be collected, including age, sex, smoking status, past medical history, family history of cancer, radiological reports, tumor markers, pathology reports and postoperative TNM-staging.

### Sample collection

20 mL blood samples will be taken by intravenous puncture at different time points. Patients recruited to this study will have their blood samples taken for at least 3 times (Blood Sample A: Before surgery; Blood Sample B: Three days after surgery; Blood Sample C: One month after surgery). Patients whose blood samples are not gathered for any of these 3 time-points will be excluded for analysis. During the follow-up period, recruited patients’ blood samples will also be gathered when thoracic CT scans are performed.

Tumor samples (Sample T) will be collected during surgery after intraoperative frozen section proves to be malignant (NSCLC). To analyze the methylation characteristics of normal lung tissue of NSCLC patients, two samples of tumor-adjacent normal tissues will be collected separately. We will collect normal tissues which are 2 cm (Sample N2) and 5 cm (Sample N5) away from the edge of tumor. Sample N5 will not be mandatory, for some patients may have wedge resection and the surgical specimens are relatively small. All the tissue samples will be stored at − 80 °C until analysis.

### Plasma collection and cfDNA isolation

Whole blood samples (20 ml) from NSCLC patients will be collected in Cell-Free DNA BCT tubes (21,892, Streck) and processed within 24 h. The temperature for transportation will be maintained at 15–35 °C according to the manufacturer’s protocol. Plasma will be extracted by centrifugation for 10 min at 2200 x g followed by 15 min at 16,000 x g using a refrigerated centrifuge. Following centrifugation, the supernatant will be transferred into a clean polypropylene tube immediately and stored at − 80 °C for future analysis. Cell-free DNA will be extracted using the QIAamp Circulating Nucleic Acid kit (55,114, Qiagen). The concentration and size profiles of isolated DNA will be examined by Qubit 3.0 dsDNA HS assay (Thermo Scientific) and HT DNA high sensitivity Labchip (Perkin Elmer). All procedures will be performed according to manufacturer’s protocols.

### DNA library preparation and sequencing

**Fig. 2:**
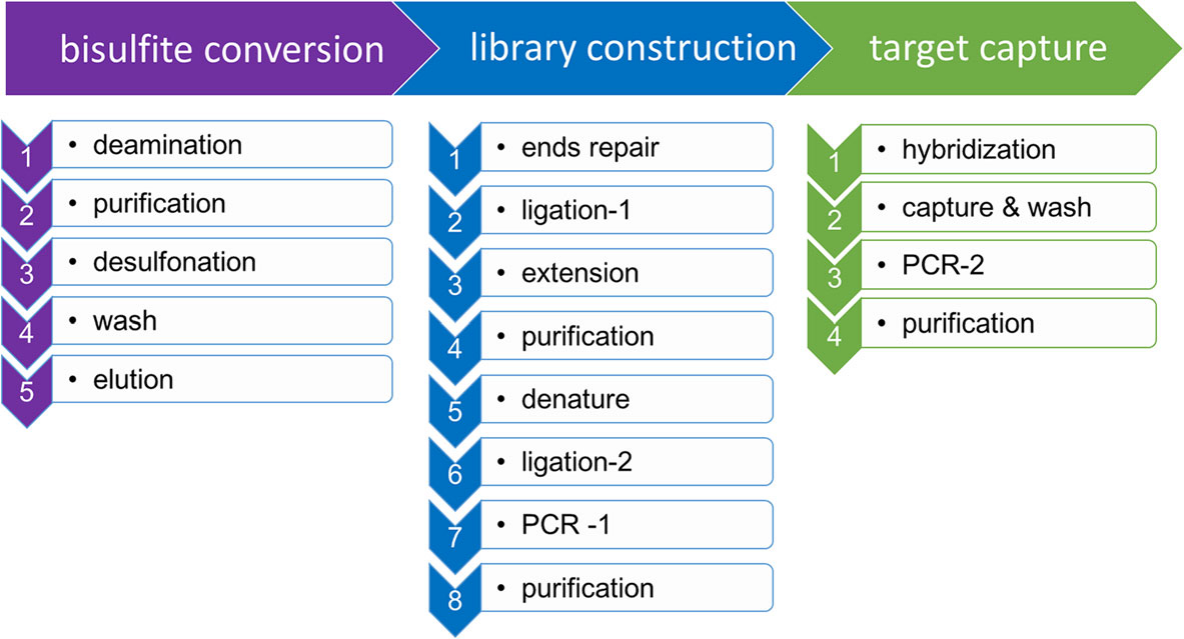
Strategy for DNA library preparation and sequencing

The brELSATM library prep method will be used to create bisulfite sequencing library illustrated in Fig. 2. Briefly, extracted cfDNA will be treated with sodium bisulfite (D5046, EZ-96 DNA Methylation-Lightning™ MagPrep, Zymo Research), turning all cytosine to uracil while leaving 5-methylcytosine unchanged. Subsequently the converted single-strand DNA molecules will be ligated to a splinted adapter, and copies of the template strands will be generated in the presence of extension primers and a uracil-tolerating DNA polymerase. After a second round of adaptor-ligation to the copy strands, 10–14 cycles of PCR reactions will be applied to obtain whole-genome BS-seq libraries. Custom-designed lung-cancer methylation profiling RNA baits will be used for target enrichment. Following a 12–16 h hybridization step, biotinylated RNA probe-bound library fragments will be selectively enriched and amplified with 14 PCR cycles. The target libraries will then be quantified by real-time PCR (Kapa Biosciences) and sequenced on NovaSeq 6000 (Illumina) using 2 × 150 bp cycles.

### Sequencing data analysis

Custom adaptor sequences and low-quality bases will be removed by trimmomatic (v0.32). BWA-meth (0.2.2) will be used to align paired-end reads to CtoT and GtoA transformed hg19 genome respectively. After alignment, PCR duplicates will be marked by samblaster (v0.1.20), and reads with low mapping quality (MAPQ< 20) or paired improperly will be removed by sambamba (0.4.7) from further downstream analyses. Merge of paired reads sequence will be performed using in-house scripts, in which overlapping reads will be clipped to avoid double-counting of methylation calls.

### Sample size calculation

According to previous studies, the sensitivity of ctDNA methylation detection is above 70% in stage I lung adenocarcinoma [21]. So we assume our panel has a sensitivity of at least 70% in stage IA to III surgical patients. As our study focuses on early stage patients, we hypothesize the postoperative positive rate to be 15% during the 3-year follow-up, and assume a 85% follow-up rate. In the field of breast cancer and colon cancer, the recurrence rate within one year was over 50% in ctDNA-positive patients and less than 10% in ctDNA-negative patients [5, 7]. We assume ctDNA have similar prognostic prediction value in NSCLC patients. Given the information above, we plan to recruit 200 individuals for the final analysis. As a result, when alpha = 0.05, the two groups (postoperative positive vs. postoperative negative) would demonstrate statistical difference on DFS, with a power over 90%.

### Data analysis plan

Clinical pathological features and other information of recruited patients will be updated every day, and will be re-checked weekly by two thoracic surgeons. All the patients who meet the requirement of follow-up and sample collection will be included into the statistical analysis. Specific reasons will be recorded for patients who are excluded.

For statistics analysis, continuous variables will be expressed as a mean ± SEM (Standard Error of Mean), while categorical variables will be coded as analyzable forms. Kaplan–Meier method will be used to estimate the distributions of disease-free survival (DFS), and the log-rank test will be used to compare the distribution of survival time. Univariate and multivariate prognostic analyses will be performed using the Cox proportional hazard model. *P* values < 0.05 will be considered statistically significant. Analysis will be performed in the R statistical environment.

### Duration of study

We designed a two-step plan for this study. We started recruitment from August 2018, and expected to successfully recruit 200 patients by August 2019. By December 2019, we plan to have generated data on all the baseline and first follow-up samples, including tissue samples (Sample T, Sample N2, and Sample N5) and blood samples (Blood Sample A to C) for ctDNA mutation and methylation detection. Meanwhile, we plan to carry out an interim analysis, including analyzing the data of ctDNA detection integrated with the corresponding clinical pathological features, comparing and associating characteristics of ctDNA methylation and mutation data among this whole cohort of early stage NSCLC patients, including but not limited to sensitivity, specificity and stability.

In the final analysis, which we expect to carry out in August 2022 with the complete follow-up data collected, the feasibility and clinical utility of ctDNA mutation and methylation detection as a means of lung cancer surveillance will be fully analyzed. As the conclusion of the study, we plan to propose a practical strategy for postoperative management of NSCLC.

This program will finish after all the patients have a 3-year follow-up.

## Discussion

Previous studies have shown that detection of ctDNA in the blood of postoperative patients is closely related to unfavorable prognosis in different tumor types, regardless of TNM staging and clinical pathological features. Postoperative patients with positive ctDNA were reported to have significantly higher recurrence rate, indicating that ctDNA may provide vital information of minimal residual disease [5, 7]. However, the detection of ctDNA in early stage lung cancer, especially adenocarcinoma, is relatively low [17]. This becomes one of the bottlenecks for the application of ctDNA currently.

On the other hand, DNA derived from tumor cells contains epigenetic information that can be detected in blood samples. Several studies have focused on the methylation of tumor tissue DNA and shown it can be used as a method of diagnosis and prognostic prediction. Aberrant DNA methylation can be detected in histological negative lymph node, and is related to poor prognosis, indicating some minimal residue disease (MRD) can be detected by this method [20]. However, detection of methylations of ctDNA has not been well established for clinical application yet. Most previous studies focused on aberrant methylations in tumor tissues, and used different panels of promoter sites. Some studies tested serum samples rather than plasma samples, indicating there still exists inconformity of specimen processing [6, 21]. Moreover, most of the previous researches were retrospective studies, which may cause insufficient assessment of the prognosis outcome and restricts the application of this method. For surgical lung cancer patients, the rationality, practicability, and stability for detection of aberrant ctDNA methylations are not illuminated systematically. And the optimal panel for NSCLC patients is still unclear.

This is the first registered study designed to prospectively evaluate and compare the detection of aberrant methylation and mutations in ctDNA among stage IA to III surgical NSCLC patients for the surveillance. The fundamental purpose of this study is to investigate the feasibility of ctDNA methylation and mutations detection as a means of lung cancer surveillance. Additionally, by analyzing the plasma samples before and after surgery, we can compare and associate biological characteristics of ctDNA methylation and ctDNA mutations. With the 3-year follow-up data, we will be able to compare the sensitivity, specificity, stability and clinical utility of the two methods, and thus propose a multi-omics information based follow-up strategy.

We expect this study can define an effective strategy for postoperative surveillance of lung cancer, by integrating liquid biopsy with current clinical practice. This may renew our knowledge of the high risk postoperative populations, and may provide new indications of adjuvant therapy [23]. However, large-scale prognostic clinical trials will be required before liquid biopsy based adjuvant therapy coming into clinical practice. In addition, this is a single institutional clinical study with a relatively long follow-up period. As a result, selection bias as well as follow-up bias may exist. Future investigations may provide complementary information of different populations.

## Data Availability

Unpublished data will be available from the corresponding author on reasonable request.

## Abbreviations

CA-125: Carbohydrate antigen 125
CA-199: Carbohydrate antigen 199
CEA: Carcinoembryonic antigen
cfDNA: Cell free DNA
CT: Computed Tomography
ctDNA: Circulating tumor DNA
CYFRA21-1: Cytokeratin 19 fragment
DFS: Disease-free survival
IL-6: Interleukin 6
MRD: Minimal residual disease
MRI: Magnetic Resonance Imaging
NSCLC: Non-small cell lung cancer
NSE: Neuron-specific enolase
PET: Positron Emission Tomography
pGGO: Pure ground glass opacity
SEM: Standard Error of Mean
